# Implementation of the Principles of Family Medicine in Modern Family Medicine Education Needing System-Specific Approaches

**DOI:** 10.7759/cureus.31177

**Published:** 2022-11-06

**Authors:** Ryuichi Ohta, Chiaki Sano

**Affiliations:** 1 Community Care, Unnan City Hospital, Unnan, JPN; 2 Community Medicine Management, Shimane University Faculty of Medicine, Izumo, JPN

**Keywords:** general medicine, rural, family physicians, nine principles of family medicine, primary healthcare, family medicine

## Abstract

Family medicine is a key specialty in primary healthcare worldwide. Medical students and residents learn family medicine in medical schools and hospitals. Modern family medicine education curricula have changed because of the increase in the number of organ specialists. Family physicians have more comprehensive training in patients, medicine, and social issues than in the past. Family physicians need to adapt to changing circumstances, but he needs to practice comprehensive and holistic medicine, as most of the time, family physicians are the first point of contact both in urban and rural setup. In 1979, McWhinney proposed nine principles of family medicine to which family physicians should adhere when providing care to patients in their communities. A review of and reflection on these principles has clarified that the principles should be more emphasized and practiced. Besides, respectful approaches by different family physicians are essential. To adapt to changing healthcare conditions, family medicine education can focus more on person-centered care and healthcare systems as system-specific specialists, respect the differences in healthcare provision between urban and rural areas, and motivate medical students and residents to become family physicians.

## Introduction

Family medicine is a key specialty in primary healthcare worldwide [1]. To improve patient-centered and community care, medical students and residents have begun to learn family medicine in medical schools and hospitals [1]. Family medicine has many principles in clinical practice [2]. McWhinney’s principles of family medicine are one of the important tenets taught worldwide in family medicine [3]. Many family physicians may adhere to these principles, also used as basic tenets in family medicine education [4]. In modern family medicine education, medical students and residents are taught the importance of person-centeredness and comprehensive approaches to patients, families, and communities.

Modern family medicine education has changed because of an increase in the number of organ specialists. Medicine has advanced, and many drugs and procedures have been developed [5]. Treatment is complicated, especially in older patients. Multiple interventions induce new medical issues, such as multimorbidity, polypharmacy, and fragmentation of care [6]. Family physicians need to adopt a more comprehensive approach to patient care and have a greater awareness of medical and social issues than in the past [7]. Family physicians also experience greater workloads than in the past. They have needed to change their specialty to become system-specific specialists by expanding the principles of family medicine. System-specific approaches have two dimensions of person and healthcare. Family physicians in this complicated medical condition have to change their perspectives from person to healthcare systems to perform comprehensive care. Family physicians deal with each patient as a system of the biopsychosocial models. Besides, they can deal with healthcare involving their patients as a system, facilitating patients’ encounters, and collaborating with other healthcare professionals and stakeholders [7]. However, there are few reports focusing on the implementation of the principles into modern family medicine conditions. Therefore, in this report, we reflect on McWhinney’s suggested principles of family medicine in the present era and discuss the concrete implementation method of the principles.

## Technical report

McWhinney proposed nine principles of family medicine that family physicians should adhere to when providing care to patients in their communities [4]. These principles have long been taught in family medicine. From the standpoint of system-specific specialists for persons and healthcare, we reflected on these nine principles [7]. 

1. Family physicians are committed to the person rather than a particular body of knowledge, group of diseases, or special techniques. Commitment is open-ended in two ways [4].

Each patient had different needs to address varying symptoms and diseases. Japanese society is aging steadily, and hospitals are crowded with older patients with multimorbidity [8]. Each person's socioeconomic status and cultural background affect their health. Family physicians have to deal with various health problems based on these contexts [8]. In such situations, family physicians are committed to each person’s health issues by listening to their needs, considering their background, and managing challenges within their contexts, termed “person-centered care.” Person-centered care is vital, even in modern family medicine. 

Family physicians need to be more comprehensive regarding managing patients’ conditions as system-specific specialists. As this principle shows, open-ended approaches are vital to dealing with complicated problems among older patients. Systems as a person can always change because a person is affected by their surroundings. Especially older people can be dependent on their surroundings because of their frailty. Family physicians need to be flexible in any challenging situations among older patients.

2. The family physician seeks to understand the context of the illness [4].

Understanding the context of patients is beneficial to their care, as contexts affect health management. For example, when family physicians begin work in a particularly rural location, they are often initially unable to understand patients’ eating habits. These family physicians are unaware of the culture of the rural island but later realize that these sociocultural factors impact people’s health [9]. Rural islands are often affected by various natural disasters, such as typhoons, so vegetables frequently cannot be cultivated over the summer months. Therefore, islanders cannot eat vegetables because of their scarcity and high cost. After understanding the specific context, family physicians can control their negative feelings toward patients and engage in dialogue with them about practical ways to manage their chronic diseases [9]. Continuing family medicine practice requires understanding the local context to mitigate conflicts and negative relationships in practice, leading to the provision of tailored medical care to patients [10]. 

Family physicians should understand patients’ contexts as one of the factors of the systems of patients. A system-specific specialist can deal with patients' conditions, including their lives and relationships with their surroundings. Patients as a system can be affected by them continuously. Family physicians can explain the change in patients’ conditions from medical science and patients’ systems. In modern family medicine education, the continual change in patients' conditions should be taught in the framework of the system.

3. The family physician sees every contact with his or her patients as an opportunity for the prevention of disease or the promotion of health [4].

This principle is beneficial for family physicians that are learning about preventative medicine and health promotion. Preventative medicine can be taught in the clinical context through interactions with various patients [11]. Patients with various symptoms and backgrounds visit family physicians. Some patients may not regularly present with chronic diseases, but they may present with hypertension and other risk factors, such as tobacco and alcohol addiction or consumption. Family physicians can identify and manage various chronic conditions in addition to addressing acute symptoms [11] through initial treatment of the latter and follow-up. These approaches can lead to both primary and secondary prevention. In family medicine departments, students learn the concepts of health promotion and maintenance through various patient encounters.

Disease prevention and health promotion should be based on a system of patients. Patients' health is sustained by the various support of healthy behaviors and regular check-ups by family physicians. Prevention and promotion impinge on a person, and other factors relating to a system in health in a person may be affected, deteriorating health conditions of a person. In modern medicine, there are more affecting factors to patients’ health conditions than in the past because of expanding the information of health even though it may be correct or not. Family physicians should be good at them in depth.

4. Family physicians view their practice as a population at risk [4].

This principle can be applied in family medicine in the modern context. Most people use primary care clinics or local hospitals, where family physicians can understand the various health issues affecting their community [12]. Patients who visit primary care medical institutions may have risk factors impacting their health conditions. Family physicians can take note of local health issues in communities through dialogue with their patients. Understanding local health issues in communities contributes to the practice of health promotion and disease prevention by family physicians [13].

Family physicians, as system-specific specialists, should broaden their perspectives from person to community. Family physicians can predict health conditions in their communities by approaching health problems in their medical institutions. Focusing on risk factors they can perceive for improvement may help people in communities change their lifestyles, eventually leading to community health. In this principle, family physicians should have broad perspectives as professionals, motivating medical students to become family physicians as specialists with a broad scope of practice.

5. Family physicians see themselves as part of a community-wide network of supportive healthcare agencies [4].

In community care, family physicians can serve as a hub for medical information related to health issues. Family physicians have to discuss local health issues with people in their communities to provide better healthcare in that specific context [14]. Collaboration between family physicians and other healthcare professionals can help develop ways to implement various strategies to solve local health issues [14]. In family medicine education, family trainees have opportunities to facilitate ways to solve local health issues through interprofessional and patient collaboration [15].

Family physicians should acknowledge that they are one of the factors in the healthcare system. Their activities can affect other factors in healthcare systems. Their attitudes to other professionals and patients change their perception regarding medicine. Family physicians’ positive attitudes can motivate them to behave productively for better healthcare. Family physicians need to be aware of being one of the stakeholders in healthcare and their influence on communities. 

6. Ideally, family physicians should share the same habitat as their patients [4].

This principle is controversial at present. Resident family physicians often live in urban locales and commute to clinics in different areas [16]. Therefore, this principle can be useful for family physicians working in rural contexts to understand the communities and cultures within their practice [17]. Although it is not necessarily critical for family physicians to live in the communities where their medical institutions are located, it can increase opportunities to engage with local people and participate in local events, which can familiarize family physicians with communities. Familiarity with communities may help them control negative feelings toward their patients [9]. In particular, rural solo family physicians will understand this principle’s importance and the critical nature of managing their emotions and patient relationships in their practice.

Wherever family physicians live, family physicians’ practice is affected by various local factors in healthcare systems. Family physicians should understand the culture and patients’ characteristics to perform effectively in communities. Healthcare as a system differs because of culture and context. In the present era, multicultural societies are advancing, and family physicians should be culturally sensitive and modify their practice to a system. Family medicine education needs to consider varieties of learning in communities among medical students. 

7. The family physician sees patients in their homes [4].

Home visits are vital for family physicians to provide palliative care to patients. Home care can enable vulnerable older patients to live in their homes in aging societies. To improve home care, family physicians must collaborate with various medical professionals, such as care managers, nurses, and care workers [17]. To facilitate this, family physicians need to communicate with patients and their families regarding the continuity of home care, as this can be a burden for families and caregivers. Therefore, family physicians need to demonstrate various communication skills to elicit ideas and opinions from stakeholders involved in home care [17]. This principle is more relevant in the modern medical context than ever before, as an aging population means that greater numbers of older people will require home care.

To be functional in home care, family physicians should work in various situations of the healthcare system in their careers. Home care needs various collaborations with professionals and organizations such as governments, hospitals, and communities. In family medicine training, family physicians can work in multiple places to understand healthcare systems. Understanding healthcare systems can facilitate family physicians to effectively care for home care patients by getting many kinds of help.

8. The family physician attaches importance to the subjective aspects of medicine [4].

This principle is a part of the three-step diagnosis process suggested by McWhinney. This consists of medical diagnosis, illness analysis, and ecological diagnosis. This principle is related to illness and ecological diagnosis [18]. Understanding these aspects enables family physicians to comprehensively analyze patients, satisfy their true needs, and prevent the progression of symptoms and diseases. Family physicians can truly empathize with patients based on their specific contexts and backgrounds.

A person as a system is affected by multiple factors in the system, but the decision must be based on the person's subjectivity. Family physicians should understand that the value of patients is one of the core factors of a person’s system. Multiple contextual factors such as families, culture, and social issues affect patients' care. In the decision-making of patients’ care, family physicians, as system-specific specialists, should respect patients' subjectivity and go along with them in their decision processes.

9. The family physician is a manager of resources [4].

This principle is vital for future family physicians who manage multimorbidity in various patients. Multidisciplinary teams often collaborate to manage multimorbidity in patients. Family physicians should have comprehensive knowledge of all aspects of patient care because specialists in multidisciplinary teams tend to focus on one aspect of treatment. Healthcare resources are limited and need to be used effectively for each patient. Specialists can drive the excessive use of resources. Family physicians should prevent excessive costs and practice healthcare sustainably.

Person as a system and healthcare systems may become complicated and chaotic without managers and leaders understanding the systems from multiple standpoints. Family physicians can potentially be managers and leaders in the systems as system-specific specialists. Family physicians should be trained in multiple clinical and cultural settings through comprehensive family medicine education. Through these experiences, family physicians could become leaders and managers of healthcare systems.

## Discussion

This technical report highlights the importance of the principles of family medicine in the modern healthcare context. As societies change, family physicians have to change their practice from diagnosis to comprehensive community care [5]. In accordance with the principles of family medicine, family physicians should respect patients' subjective backgrounds and consider that their patients’ daily lives and habits can be related to their diseases [4]. In addition, each patient has different help-seeking behaviors (HSB) [19]. Family physicians can consider each patient to be an individual with different ways of approaching their symptoms and help them to achieve alternative HSB [4]. Investigating patients’ experiences of their symptoms, which are sometimes independent of physical findings, can occasionally lead to a diagnosis. 

At present, the working styles and settings of family physicians vary, and their scope of practice depends on their work situation. Rural physicians are often older, while younger family physicians tend to work in urban areas. Their residences can differ from their professional contexts [16], as some family physicians commute to work from urban to rural areas. Each family physician’s working style should be respected based on changing healthcare conditions. In addition, differences in the scope of practice in urban and rural areas can affect family physicians’ motivation in their workplaces [20]. In rural contexts, family physicians should deal with a large variety of medical and psychosocial problems, so they often need a wider range of scope of practice than is required in urban areas [20]. In addition, healthcare resources are often limited in rural contexts; rural family physicians, as resource managers, should carefully diagnose patients based on their clinical history and physical examinations and by honestly listening to their patients [4]. On the other hand, as there are many specialists in urban areas, urban family physicians need to effectively collaborate with various specialists, requiring more collaboration skills. Therefore, medical students and family medicine residents can choose their work settings based on their interests and advantages.

Considering system-specific specialists' framework, these principles can lead to the advancement of the specialty of family physicians in modern healthcare. The first to third principles focus on a person's education as a system. Through learning these principles, family physicians become specialists in person-centered care. Forth to sixth principles broaden family physicians' perspectives from the individual to healthcare systems. Through learning these principles, family physicians could be a specialist familiar with public health and sociology, respecting culture and societies. Finally, the seventh to ninth principles teach family physicians the importance of learning respect for individuals and working in multiple settings, leading to leaders and managers in healthcare systems. Deep learning in family medicine principles can make modern healthcare systems more efficient and effective.

## Conclusions

Family medicine principles must be applied to family medicine education, respecting the change in healthcare conditions in each practical situation. In changing practical situations, family physicians are now forced to practice not system-specific or comprehensive but super specialty-centric approach. Family physicians may tend to become a reference point. Systems approaches by family physicians are essential for patient-centered care and comprehensive care. Family medicine education should focus on system-specific specialties and comprehensive care to adapt to changing healthcare conditions. Furthermore, the differences in healthcare provision between urban and rural areas should be respected, and medical students and residents can be motivated to learn system-based and comprehensive care as a specialty of family medicine.
